# CoSiM-RPO: Cooperative Routing with Sink Mobility for Reliable and Persistent Operation in Underwater Acoustic Wireless Sensor Networks

**DOI:** 10.3390/s19051101

**Published:** 2019-03-04

**Authors:** Munsif Ali, Anwar Khan, Khursheed Aurangzeb, Ihsan Ali, Hasan Mahmood, Syed Irtaza Haider, Naeem Bhatti

**Affiliations:** 1 Department of Electronics, Quaid-i-Azam University Islamabad, Islamabad 45320, Pakistan; mali@ele.qau.edu.pk (M.A.); hasan@qau.edu.pk (H.M.); nbhatti@qau.edu.pk (N.B.); 2 Department of Electronics, University of Peshawar, Peshawar 25120, Pakistan; 3 Computer Engineering Department, College of Computer and Information Sciences, King Saud University, Riyadh 11543, Saudi Arabia; kaurangzeb@ksu.edu.sa (K.A.); sirtaza@ksu.edu.sa (S.I.H.); 4 Department of Computer System and Technology, Faculty of Computer Science and Information Technology, University of Malaya, Kualalumpur 50603, Malaysia; ihsanalichd@siswa.um.edu.my

**Keywords:** UA-WSNs, sink mobility, routing/algorithm, performance improvement

## Abstract

An efficient algorithm for the persistence operation of data routing is crucial due to the uniqueness and challenges of the aqueous medium of the underwater acoustic wireless sensor networks (UA-WSNs). The existing multi-hop algorithms have a high energy cost, data loss, and less stability due to many forwarders for a single-packet delivery. In order to tackle these constraints and limitations, two algorithms using sink mobility and cooperative technique for UA-WSNs are devised. The first one is sink mobility for reliable and persistence operation (SiM-RPO) in UA-WSNs, and the second is the enhanced version of the SiM-RPO named CoSiM-RPO, which utilizes the cooperative technique for better exchanging of the information and minimizes data loss probability. To cover all of the network through mobile sinks (MSs), the division of the network into small portions is accomplished. The path pattern is determined for MSs in a manner to receive data even from a single node in the network. The MSs pick the data directly from the nodes and check them for the errors. When erroneous data are received at the MS, then the relay cooperates to receive correct data. The proposed algorithm boosts the network lifespan, throughput, delay, and stability more than the existing counterpart schemes.

## 1. Introduction

Persistence operation of UA-WSNs needs scrutiny due to its applications, uniqueness, challenges, and difficulties. Applications like submarine detection and navigation, surveillance, marine animal imaging, oil spill detection, pollution monitoring, etc. [1,2,3], require such an algorithm for their persistence operation. It is important because of the numerous hindrances and challenges. UA-WSNs are more challenging because of the harshness of the water medium [4]. The acoustic waves are used for communication instead of the radio and optical waves. This is due to the absorption and attenuation in the water of the radio waves [5]. On the other hand, the optical waves are scattered extremely in the water. Therefore, priority is given to the acoustic waves for communication underwater. However, these waves have a much slower speed than the radio and optical waves, which brings delay in the packet delivery [6]. Furthermore, several factors influence the acoustic waves such as noise, attenuation, Doppler spread, and multi-path fading [7,8]. It is, therefore, required to have an efficient mechanism to deliver data with a minimum latency and high accuracy due to the unpredictable nature and high bit error rate (BER). Moreover, energy-efficient algorithms are required because of the limited energy source of the nodes, and it is tough to replace or re-energize the battery [9]. Furthermore, GPS is required for location information acquisition, which is difficult underwater [10]. Many other techniques have been proposed that use optical waves for localization of the network [11,12]. However, the optical waves are used for short distance communication. Likewise, the water currents change the position of the nodes and randomly move in the network [13]. Therefore, determining the exact position is a difficult task. Moreover, an energy-efficient and low-cost modem is required for the UA-WSNs’ communications [14]. Furthermore, the ocean covers 70% of our planet, and most of the area has almost been unexplored even after much research [15]. Due to the aforesaid applications, reasons, and challenges, it has attracted the attention of researchers, academia, governments, and engineers.

Data exchanging is accomplished in two ways in UA-WSNs. The first is that in which the data are transferred through a single path without the help of relays to the desired destination, known as the non-cooperative scheme. While in the second case, the relays assist in more accurate data reception, termed cooperative algorithms [16]. Due to data exchanging through a single path compromised of the data loss probability in a non-cooperative algorithm, using cooperative techniques provide reliable data exchanging by utilizing broadcast nature. In cooperative routing, the relays work together with the sender to deliver data more accurately to the destination. The relay assists in two ways: in the first, which is specified as fixed cooperation (FC), the data delivery to the destination is accomplished as it receives data from the sender without any conditions; in the second case, named incremental cooperation (IC), the relay waits for the signal reception from the destination [17]. After signal reception, the data are delivered by the relay. The data forwarding through a relay is done in two ways. The relay either boosts the received data, named amplify and forward (AF), or fully decodes them, titled decode and forward (DF) [18]. Due to the simplicity of the AF scheme, it is mostly used instead of DF. The cooperative algorithms enhance the chances of successful data delivery and compromise on the energy cost and delay.

The energy cost, latency, and data loss probability of the multi-hop algorithms [19,20,21,22] are high and have less stability due to the involvement of many nodes for a single-packet delivery. Moreover, performance decreases as holes (void regions) are created due to the death of the nodes. A node stuck in a void region is useless because of no link available to the surface. The multi-hop cooperative routing [23,24,25,26] provides high data accuracy and less packet dropping due to the exchanging of a single packet through many paths. However, the remaining aforesaid issues in multi-hop algorithms increase to a high extent in cooperative algorithms. On the other hand, the routing algorithms with sink mobility provides persistent operation of the network. The mobile sinks (MSs) try to reach every node to collect data from it directly to gain better throughput with less resources, which overcomes the concerned challenges of the multi-hop algorithms. In the existing algorithms with sink mobility and cooperation in [27,28], the MSs do not cover all the network and still require multi-hop routing. Furthermore, data forwarding in a cooperative manner leads to an increase in the energy cost of the network. The algorithms presented in [29,30,31] has no wait and data holding mechanism, and the data are dropped when an MS is not in the coverage of the node.

To address the above-mentioned limitations of the existing algorithms, this paper presents an algorithm with sink mobility exploiting cooperation for UA-WSNs. First, the network is partitioned into four equal squares. Four MSs move continuously in these squares to report data from the nodes. The direct link is established for data exchanging between an MS and a node in the case of MS presence in the coverage area of the node. Otherwise, the node holds data and waits for the MS to reach it. For the coverage of all of the network, a triangular path is determined for the MS. Furthermore, the MS not only reports data from specific regions’ nodes, but also, it can collect data from any region that it covers. The data are determined for acceptance at the MS if they hold the specific criteria of the data correction. Otherwise, the MS demands the retransmission of the packet. Upon the demand, the relay cooperates with the MS for successful data delivery. Our contribution is summarized as:

- The proposed SiM-RPO algorithm tends to perform persistent operation of the network and achieves high throughput with minimum energy cost and latency. For the sake of MSs’ path determination and their collision avoidance with each other, the network is partitioned into four regions having equal area. The MSs move in their own regions on the determined path to pick the information from the nodes directly. The path is determined for all the MSs in such a way to so as cover all the network and pick the data even from a single node.
- Furthermore, to reduce the latency and energy expenditure, the data are delivered to the MS directly instead of multi-hoping. The data are delivered directly to the MS when a node finds the MS in its coverage area. Otherwise, the node waits for the MS and holds the data.
- In SiM-RPO, delivery over a single path does not ensure successful transfer of the information. Therefore, the addition of cooperation to the SiM-RPO, titled CoSiM-RPO, boosts the reliability and data reception probability. In CoSiM-RPO, the data are analyzed for errors at the MS for satisfied data reception. The data are considered correct if they hold the specified criteria of the BER. Otherwise, the MS demands data retransmission from the relay. The data is transferred again by the relay to the MS.

The arrangement of the paper is as follows. The main idea of the existing approaches is described in Section 2. The proposed algorithms SiM-RPO and CoSiM-RPO are presented with the necessary detail in Section 4 and Section 5, respectively. In Section 6, the MATLAB simulation of both the proposed schemes compared with the three existing algorithms is given with the full description. The last section presents the conclusions and future insights.

## 2. Literature and Background of UA-WSNs

Sajid et al. proposed a cooperative algorithm with sink mobility for UA-WSNs in [28]. Two sinks move linearly in the middle of the network and gather the information and data from the nodes. The node forwards data in a multi-hop fashion with the help of relay when the MS is not in the coverage area of the node. When the MS is present in the coverage area of the node, then it delivers the data directly to the MS. The efficiency of the network is improved such as the lifespan and minimal packets being dropped compared to its counterpart schemes. However, due to the cooperation of nodes, the latency and energy cost of the network are high.

Another idea that uses two MSs is presented in [29]. The circular regions are defined in the network. In these circular regions, the MSs move in a clockwise direction. The nodes deliver data and information directly to the MSs when they come in the nodes’ transmission range. This arrangement gains better lifetime and delivery to the base station. However, in this method, the packet is dropped in case of the absence of the MS in the coverage area of the node. The same idea of the data forwarding mechanism is followed in [30]. However, the network division is done in ten rectangular sectors. Two MSs pick up the data from the nodes. The algorithm gains better lifetime, throughput, and stability.

The authors in [32] proposed an algorithm in which the network is split into three small horizontal sectors. Two MSs move in the horizontal orientation, whereas two MSs in the vertical orientation in the network. The MS receives data directly from the node when the MS is in the coverage area of the node, otherwise cooperative routing is performed. For further improvement and to operate the network for a greater time, energy-harvesting techniques are utilized. This approach improves the lifetime and throughput of the network. However, this approach still uses multi-hop communication, which leads to deliver data with latency.

Yahya et al. proposed an algorithm for UA-WSNs with sink mobility in [33]. The network is split into two vertical sectors. Further, each vertical sector is partitioned into five sectors. Overall, ten sectors are formed in the whole network. Two MSs move in their own vertical region for information collection and one static sink fixed at the network top center position. The MSs do not follow a specific path, but they search for the sparse regions and move to the sparse regions for data collection from the nodes. The nodes in the dense area communicate with the cluster head. Then, it advances data to the MSs or to the base station. The throughput and network life period are maximized in this scheme.

A MobiSink protocol with cooperation mechanism is presented by Shah in [34]. Four horizontal sections are formed in the network, and four MSs move horizontally in each section of the network. The direct data reception is done when the node in its coverage area finds an MS. Otherwise, the data are delivered to the MS through a neighbor node. MobiSink achieves better throughput by paying a greater cost of power of the nodes.

In [35], Akbar et al. presented a routing mechanism for a 3D network that uses the MS and cluster nodes. The MS and cluster nodes move in the defined region formed in the network for data collection from the nodes instead of multi-hop data forwarding to improve the network lifetime, throughput, and path loss. If a node has an MS in its coverage area, then it forwards data to the MS. Otherwise, in the absence of the MS, the data are received by the cluster node and then proceeded to the MS.

The algorithm presented in [36] partitioned the whole network into four small squares. Instead of static sinks, two MSs are used in the network. The nodes search for an MS in proximity to deliver data. The direct exchange of the data is accomplished when the MS is present in the proximity of the node. While in absence of the MS, the data are discarded. This achieves a high delivery of the packets at the final station with a minimum cost of energy and improves the lifespan of the network.

In [37], the authors established the network as ten small portions. Two MSs are considered to collect all the information sent by the nodes. If a movable sink is available in the coverage area of the node, it collects data directly. Otherwise, the destination is selected for the data delivery to the MS. The destination checks the erroneous bits in the data when it receives the data and makes a decision for the acceptance or rejection of the data. When an MS receives data, it moves forward in the direction of the other nodes to collect data in order to increase delivery. The algorithm improves the packet delivery ratio (PDR) and consumption of energy.

An opportunistic routing with void avoidance using MSs is presented in [38]. The division of the network into small cubes makes the energy expenditure better. The MSs pick the data from the nodes and also from the void area nodes to prevent data failure. The nodes hold the data for a predefined time for better packet reception. The performance of the network in terms of energy cost, delay, and throughput are enhanced.

A receiver-initiated MAC is presented in [39] by Yuan Dong. The protocol consists of four phases using three-way handshaking. In the first phase, the receiver sends the RTR (request to receive) message. Then, the sender in the second phase responds to the receiver using the ATS (available to send) message for the data transmission. Then, the sender starts exchanging of the data in the third phase. In the final stage, the receiver acknowledges reception of the data.

In [40], a MAC protocol is proposed, which addresses the hidden terminal problem, as well as the spatial-temporal uncertainty. Both the sender and receiver schedule data sending and reception. The node awakes when it has to send the data. Otherwise, the node sleeps to reduce the energy expenditure.

An energy-efficient MAC protocol is proposed in [41] using both optical and acoustic waves for communication. First, it ensures that the idle state of the channel uses the acoustic waves. If the channel is not free, the node waits. Then, for the data exchanging with optical waves, the channel is detected using the optical waves. If the channel supports the optical waves transmission, then the data are delivered with the optical waves. Otherwise, the high priority data are delivered using acoustic waves to the next destination.

A hybrid network that utilizes both the acoustic and optical waves is presented in [42]. The optical waves are used for data exchanging with a high data rate, while the acoustic waves are used for the information sharing between nodes.

## 3. Channel Model

The detailed description of the channel noise and attenuation used in this paper is discussed in this section.

### 3.1. Noise

For UWSNs, the noise sources are categorized into four classes. These noise sources are known as turbulence, shipping, wind, and thermal noise [43]. In the following equations, each noise source power spectral density (PSD) in dB re μ Pa per Hz is given as:

(1) $$\begin{array}{c} 10logN_{t}\left(f\right)=17-30log\left(f\right)\\ 10logN_{s}\left(f\right)=40+20\left(s-0.5\right)+26log\left(f\right)-60log\left(f+0.03\right)\\ 10logN_{w}\left(f\right)=50+7.5w^{1/2}+20log\left(f\right)-40log\left(f+0.4\right)\\ 10logN_{th}\left(f\right)=-15+20log\left(f\right) \end{array}$$

$N_{t}$, $N_{s}$, $N_{w}$, and $N_{th}$ represent the PSD of turbulence noise, shipping noise, wind noise, and thermal noise, respectively. The frequency *f* is measured in kHz. The shipping parameter *s* varies from 0–1 for low and high shipping, respectively. The wind speed *w* given in m/s is the dominant source of the noise. The combination of all these noise sources affects the desired signal. The overall noise PSD is given by the following equation:

(2) $$N=N_{t}\left(f\right)+N_{s}\left(f\right)+N_{w}\left(f\right)+N_{th}\left(f\right)$$

### 3.2. Attenuation

The attenuation also affects the desired signal. The attenuation depends on distance *d* from the source to the destination and on selected frequency *f*. The attenuation A(d,f) is given as [43]:

(3) $$A\left(d,f\right)=A_{o}d^{k}\alpha \left(f\right)$$

The *k* represents the spreading factor, and α(f) represents the absorption coefficient, respectively. Following is the equation to express attenuation in dB.

(4) $$10logA\left(d,f\right)/A_{o}=k.10log\left(d\right)+d.10log\alpha \left(f\right)$$

The spreading loss is represented by the first term of the above equation, and the absorption loss is represented by the second term, respectively. The *k* is taken as 1.5 for practical spreading. Using Thorp’s formula, the absorption coefficient α(f) in dB/Km for frequency *f* less than 0.4 kHz and above this is given by the following equations, respectively.

(5) $$\begin{array}{c} 10log\alpha \left(f\right)=\frac{0.11\times f^{2}}{1+f^{2}}+\frac{44\times f^{2}}{4100+f^{2}}+\frac{2.75\times f^{2}}{10^{4}}+0.003 \end{array}$$

(6) $$\begin{array}{c} 10log\alpha \left(f\right)=0.002+\frac{0.11\times f^{2}}{1+f^{2}}+0.11f^{2} \end{array}$$

### 3.3. Energy Consumption Model

The nodes consume different amounts of energy during transmission and reception of the data. When a node transmits *n* bits of data at a distance *d*, the energy $E_{tx}$ is used by the sender, which is [33]:

(7) $$E_{tx}=n\times E_{elec}+n\times T_{b}$$

where $E_{elec}$ represents the dissipated energy and $T_{b}$ is the time duration for a single bit. When a node receives data, it consumes some amount of energy $E_{rx}$, which is determined as [33]:

(8) $$E_{rx}=n\times E_{elec}$$

The initial energy of every node $E_{in}$ is dissipated after processing of the data, and the residual energy $E_{re}$ is given as:

(9) $$E_{re}=E_{in}-\left(E_{tx}+E_{rx}\right)$$

While the MS residual energy is:

(10) $$E_{re}=E_{in}-\left(E_{tx}+E_{rx}-E_{m}\right)$$

where $E_{m}$ is the consumed energy due to the movement of the MS.

## 4. Proposed SiM-RPO Algorithm

### 4.1. Network Composition

**Figure 1:**
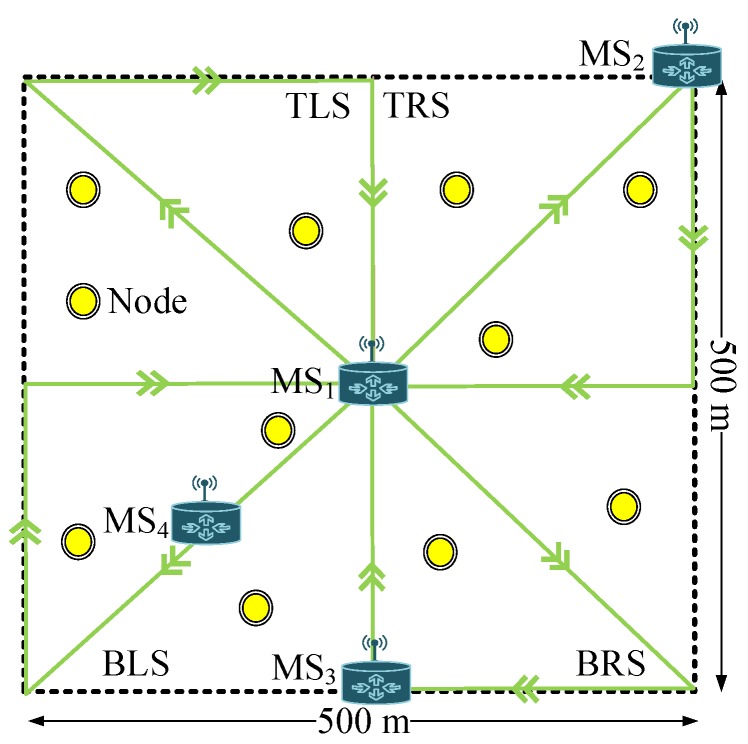
Proposed network. MS, mobile sink; TRS, top right square; TLS, top left square; BRS, bottom right square; BLS, bottom left square.

A 2D network is designed for underwater having each dimension 500 m. Further, the network is partitioned into four equal squares and named as the top right square (TRS), top left square (TLS), bottom right square (BRS), and bottom left square (BLS), as shown in Figure 1. In each portion, an MS moves in a triangular path to pick data from the nodes. Irregular deployed nodes sense the attributes and convert the sensed attributes into data packets. The generated data for further processing are delivered to the MS. The nodes communicate directly with the MSs. Communication between nodes takes place through acoustic waves, and every node is equipped with an acoustic modem. All nodes are considered homogenous in all aspects. The method of position determination for the autonomous underwater vehicle in [44] is assumed here for the MS location information. The sinks are deployed at the surface, which are equipped with GPS. These sinks provide help for determining the position of the MS. The distance from these surface sinks is calculated using the time of arrival and speed of the acoustic waves. Then, the coordinates are estimated using the relative distance from the surface sinks, which shows the current position of the MS. For example, if an MS is at the center position, then its distance from each surface sink will be the same. The ordinary nodes do not need location information in the proposed algorithms.

### 4.2. Path Determination for MS

The path determination for the MSs is designed in such a way that they cover all the network and pick more information from the nodes. A triangular path is defined for the MS, and it moves continuously on the same path. MS_1_, MS_2_, MS_3_, and MS_4_ start their motion from the center, corner, mid-boundary, and center of the BLS in their respective regions, TLS, TRS, BRS, and BLS, as shown in Figure 1. The starting point of each MS is kept different instead of the same point due to the data collision and interference. That ensures it will not come to the center at the same time. When the data are received multiple times at the surface sink, it is assumed that advanced techniques are being used by the surface sink for the extraction of the desired information. The center or middle of the network is:

(11) $$C\left(x_{c},y_{c}\right)=\frac{x_{o}+x_{f}}{2},\frac{y_{o}+y_{f}}{2}$$

where ($x_{c}$, $y_{c}$), ($x_{o}$, $y_{o}$), and ($x_{f}$, $y_{f}$) are the center, initial, and final coordinates of the network, respectively. There are three types of motion of the MS, i.e., diagonal, horizontal, and vertical. All the MSs start motion from their starting point and move in the network on the predefined path.

Mathematically, the diagonal movement of every MS by a shift of fixed quantity *z* from its present position ($x_{p}$, $y_{p}$) to the new position ($x_{n}$, $y_{n}$) is given as:

(12) $$MS_{1}\left(x_{n},y_{n}\right)=\left(x_{p}-z,y_{p}-z\right)$$

(13) $$MS_{2}\left(x_{n},y_{n}\right)=\left(x_{p}+z,y_{p}-z\right)$$

(14) $$MS_{3}\left(x_{n},y_{n}\right)=\left(x_{p}+z,y_{p}+z\right)$$

(15) $$MS_{4}\left(x_{n},y_{n}\right)=\left(x_{p}-z,y_{p}+z\right)$$

When the MSs are at the corner of the network, they start moving along the boundary of the network either in a horizontal or vertical direction to avoid collision with each other. MS_1_ and MS_2_ move horizontally and MS_2_ and MS_4_ move vertically. The next coordinates of every MS are:

(16) $$MS_{1}\left(x_{n},y_{n}\right)=\left(x_{p}+z,y_{p}\right)$$

(17) $$MS_{2}\left(x_{n},y_{n}\right)=\left(x_{p},y_{p}+z\right)$$

(18) $$MS_{3}\left(x_{n},y_{n}\right)=\left(x_{p}-z,y_{p}\right)$$

(19) $$MS_{4}\left(x_{n},y_{n}\right)=\left(x_{p},y_{p}-z\right)$$

When the MSs are at the midpoint on the boundary of the network, they start moving toward the center of the network and change their horizontal movements to vertical or vice versa, given as:

(20) $$MS_{1}\left(x_{n},y_{n}\right)=\left(x_{p},y_{p}+z\right)$$

(21) $$MS_{2}\left(x_{n},y_{n}\right)=\left(x_{p}-z,y_{p}\right)$$

(22) $$MS_{3}\left(x_{n},y_{n}\right)=\left(x_{p},y_{p}-z\right)$$

(23) $$MS_{4}\left(x_{n},y_{n}\right)=\left(x_{p}+z,y_{p}\right)$$

The path direction for all the MSs is shown in Figure 1 by a solid green line.

### 4.3. Bound MSs’ Movement within the Networks

Initially, all the MSs are deployed in a specific position in the network, and then, they start moving along with the defined path in the network. However, how does one bound the MSs movement in the network and follow the defined path? Which do not go outside of the network? When an MS reaches the position in the network where it changes movement direction, then how does it know that specific position? All the MSs move in a specific region, so the coordinates of the region at which the MSs change the movement direction are stored in the memory of the MS. Three network coordinates, center, corner, and mid-boundary coordinates, are stored. When an MS moves a step forward, then it checks its present coordinates with the stored coordinates. If it is equal to one of the stored coordinates, then it changes its movement direction and defines its direction according to the stored coordinates. Otherwise, it moves in the same direction. The addition or subtraction of a fixed quantity *z* defines the direction of the MSs’ movement. For example, MS_1_ starts its motion from the center of the network. MS_1_ checks its current position after moving to the next step with the stored coordinates. When the present coordinates do not equal any of the stored coordinates, then it moves in the same direction. When its coordinates become equal to the corner coordinates, then it increases only the *x* coordinate by adding a fixed value of *z* while the *y* coordinate remains same. The addition of a fixed value *z* to *x* indicates that the movement of the MS_1_ is in the horizontal direction and bounds the MS_1_ in the network. In another case, if the coordinates are equal to the midpoint, then it starts increasing only the *y* coordinate, which indicates the vertical movement. When the coordinates are equal to the center coordinates, then it moves in the diagonal direction. Likewise, this is done for all the MSs to follow the specified path in the network.

To track the MS on the specified path, the increment or decrement of the *x* and *y* coordinates forces the MS to follow the specified path. Changing both the *x* and *y* coordinates specifies the diagonal movement of the MS, while changing only one coordinate determines the horizontal or vertical movement of the MS. To change the direction of the MS, it periodically checks its coordinates with the center, corner, and mid-boundary coordinates of the network, which are stored in the memory of the MS. When its present coordinates are matched with the stored values, then the MS changes its direction.

The water currents can cause the MS to diverge from its path. Therefore, to keep the MS on the track, an error *e* is calculated by every MS when moving to the next step. When an MS step forwards from the current position ($x_{p}$, $y_{p}$) to the next position ($x_{n}$, $y_{n}$), then it is compared with the estimated position value from the surface sinks to confirm the actual position of the MS and detect the drifts. When an MS step forwards, then it checks its new position $\left(x_{n},y_{n}\right)$ with the previous position $\left(x_{p},y_{p}\right)$ and finds the error *e* as:

(24) $$e=|x_{p}-x_{n}|-1$$

Furthermore, for the *y* coordinates, *e* is:

(25) $$e=|y_{p}-y_{n}|-1$$

The value of *e* will be zero if the water currents do not disturb the movement direction of the MS. If *e* has some positive or negative value, then it will be subtracted or added respectively, when moving to the next position to follow the specified triangular path. In case of positive error, the next coordinates are calculated as:

(26) $$MS\left(x_{n},y_{n}\right)=\left(x_{p}\pm z-e,y_{p}\pm z-e\right)$$

In case of negative error, the next coordinates are:

(27) $$MS\left(x_{n},y_{n}\right)=\left(x_{p}\pm z+e,y_{p}\pm z+e\right)$$

For example, the MS is at the (250, 250) position, and it moves to the next position (251, 251). In this case, the water currents do not disturb the MS movement, and the error is zero. When the water currents disturb the MS position, then *e* will be some positive or negative value, which indicates that the MS deviated from its track and can be tracked back by adding or subtracting the error values when finding the next position.

### 4.4. Data Link Establishment and Exchanging

**Figure 2:**
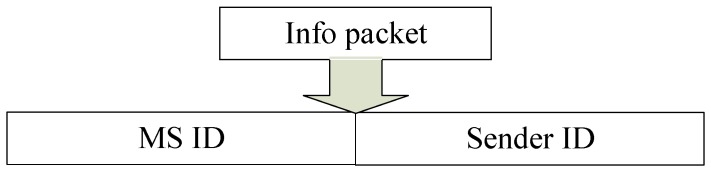
Info packet format.

The link is established before data exchanging between an MS and a node. For link establishment, the info packet is broadcast by every MS having a 130-m transmission range. The info packet encloses the ID of the MS and node as shown in Figure 2. The info packet reception by a node shows the presence of the MS in the coverage range of the node, which is 130 m. The info packet reception indicates the establishment of the link between MS and a node for data exchanging. If the info packet is received by a node, then the data are exchanged with the MS. Otherwise, the node holds data until an MS reaches that node. When an MS receives data, then it delivers directly to the surface sink node or to the next MS. MS_1_ and MS_2_ directly transmit data, while MS_3_ and MS_4_ forward to MS_2_ and MS_1_, respectively. In multi-hop transmission, there is an extra burden of a sender on the other nodes for data delivery to the surface. To reduce the extra burden on the nodes, the MSs directly receive data from the nodes and deliver them to the surface. Info packet transmission is done by the MS to save the energy of the node, because, when a node transmits the info packet, this leads to the reduction of the network lifetime. This is due to nodes having limited energy.

The MS broadcasts an info packet after some interval of time for the data exchanging with any node. When the position of the node changes with water currents and the link between node and MS breaks, then the node stops data forwarding to the MS, holds the data, and waits for the info packet reception again to exchange the data with the MS.

### 4.5. MS Prioritization and Nodes’ Coverage

**Figure 3:**
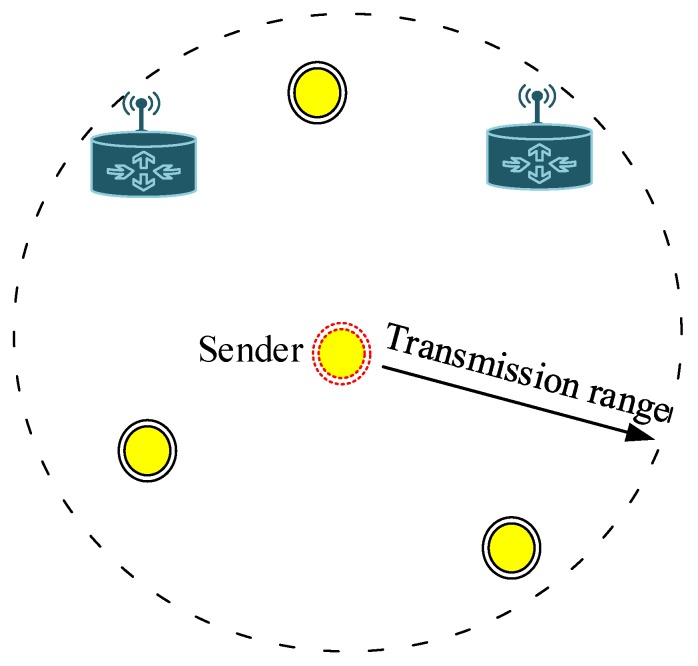
MS prioritization.

For more information and data collection, the MSs are not bounded to collect data only from their own regions’ nodes. Rather, they can collect data from any region of the network. The paths determination is done for coverage of all the network. If a node finds multiple MSs in its transmission range, as shown in Figure 3, it communicates with the MS having high priority. High priority is assigned to MS_1_ and then to MS_2_, MS_3_, and MS_4_, respectively.

If a node lies at a corner of the square opposite the triangular path, then it cannot communicate with the MS in the same square region. This is because the node having a 130-m transmission range does not hear the info packet for data forwarding from that MS. However, it communicates with other MSs in the adjacent square region. Because it hears the info packet for data forwarding from that MS, and all of the network is covered by the MSs for the collection of the data.

## 5. Cooperative SiM-RPO

In the SiM-RPO algorithm, delivery of the data through a single path has a high chance of packet loss. This is because of the underwater noisy channel, harsh environment, path losses, and attenuation. Therefore, the data reception at the MS may be corrupted and more erroneous. Therefore, for satisfactory data reception, the data are analyzed by the MS. If the data have a high BER than a defined value, then the data are retransmitted only one time on the demand of the MS by a relay. Otherwise, the data reception at the MS is considered successful.

**Figure 4:**
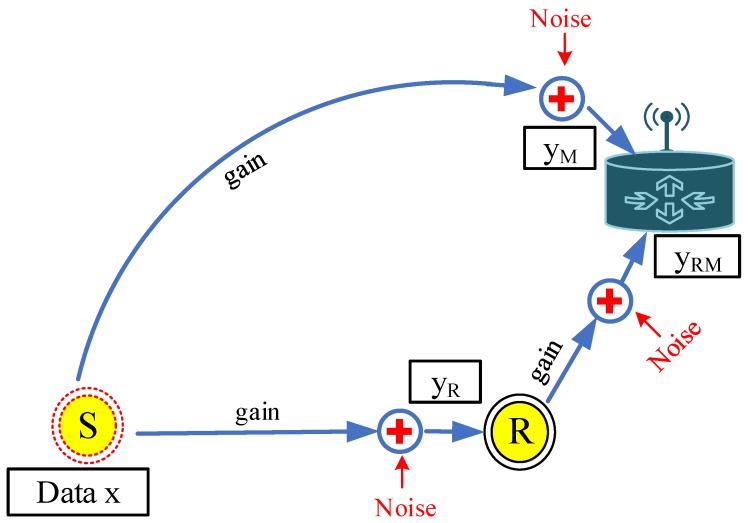
Data cooperation.

**Figure 5:**
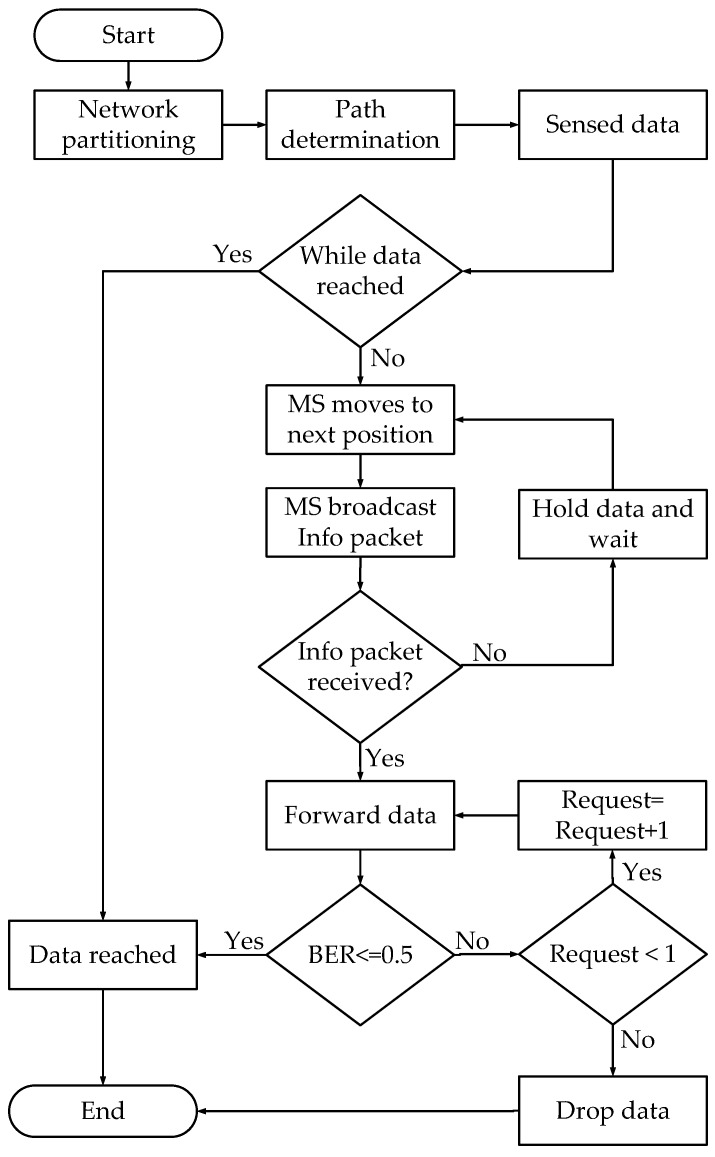
Flowchart for the cooperative sink mobility for reliable and persistence operation (CoSiM-RPO) scheme.

In the CoSiM-RPO algorithm, data exchanging of a node *S* with the MS in a cooperative manner through a relay *R* is as shown in Figure 4. When the *S* broadcasts data *x* toward the MS, then the effected data $y_{M}$ and $y_{R}$ by the channel gain *g* and noise *n* are received at the MS and neighbors, respectively. The received data $y_{M}$ at the MS are analyzed for errors. If the errors in the data are less than a defined value, it is considered successful at the MS and acknowledged by the MS. Otherwise, in case of errors greater than the defined value, a request by the MS is broadcasted. In response, the relay broadcasts its data $y_{R}$ that it received from the sender. The MS receives the relaying data $y_{RM}$ and analyzes further if they satisfy the BER threshold, and it is considered that the data are delivered successfully. Otherwise, the data are discarded by the MS. The Algorithm 1 and flowchart in Figure 5 also explain the proposed algorithm.

**Algorithm 1:** Algorithm for the CoSiM-RPO scheme.

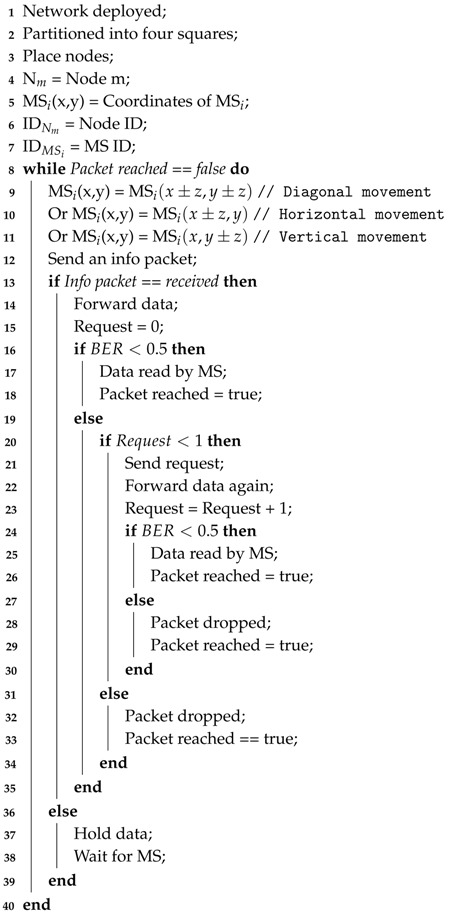



### 5.1. Relay Selection and Data Holding

When the MS requests for the data, it sends the request packet of a few bits. The nodes that hear the request packet deliver their data to the MS. However, all these nodes’ data transmission creates a data burden and excessive data forwarding. To avoid these redundant transmissions, only one relay transmits its data to the MS. The node that receives the request first broadcasts its data without any latency and embeds its own ID with the data. As the relay broadcasts data, it removes these data from its memory. The other nodes also hear the relaying data. When the nodes receive data, then they check their data saved in the memory against the received data. If both data packets have the same packet number and sender ID, then other relays discard the data from memory to avoid redundant data transmission. Every data packet has its own number and sender ID in the header of the data.

The nodes hold the data until they receive an acknowledgment or request from the MS. In the case of an acknowledgment reception, the nodes discard their data. In the case of request reception, the node that receives the request first broadcasts the data at that time and discards them, while the other nodes discard the data after reception of the relaying data. The node that does not hear the acknowledgment, request, or relaying data discards the data after some time.

## 6. Simulation Results

Performance validation of the proposed SiM-RPO and CoSiM-RPO algorithms is done through simulation using MATLAB. The proposed schemes are compared with the existing depth based routing DBR [19], Optimized DBR [20], and Cooperative DBR [23] algorithms. A network having dimensions of 500 m × 500 m is considered below the ocean surface, as considered in the existing scheme [23]. The network is partitioned into four small squares named TRS, TLS, BRS, and BLS. The MSs move on a predefined triangular path for data collection. The MSs start motion from the defined points in the network. The MS at the center point moves in a diagonal direction towards the corner of the network. When the MS reaches the corner, it moves along the *x*-axis or *y*-axis. When it reaches to the middle of the network or the other corner of the square, then it starts moving back to its start position. It makes a triangular path, covers the whole network, and obtain access to all the nodes for information and data collection. All the other MSs follow the same procedure to move in a triangular path. The irregular 225 nodes make a connection with MSs through the acoustic link. Therefore, an acoustic modem is embedded with every node. The nodes consume 2 W, 0.8 W, and 8 mW of power while transmitting data, receiving, and in the idle state, respectively. An equal amount of energy is given to every sensor node at the initial stage. The info packet size is 48 bits. The data packet size is 1600 bits and transmitted with a rate of 10 kbps. Table 1 provides a summary of the simulations undertaken.

The water flow disturbs the nodes’ position. Therefore, the nodes move randomly and are not static. Before the transmission or exchanging of the data, the MAC protocol senses the channel as used in [39]. The three-way handshaking MAC protocol is used. The MS initiates info packet for their presence, as well as an indication that the MS is ready to receive data from the nodes. When the node hears info packet, then it transfers data to the MS. When the MS receives data, then it sends acknowledgment to the sender. The data are delivered to the destination by the source if the channel is free. Otherwise, after several attempts, the data are dropped.

**Figure 6:**
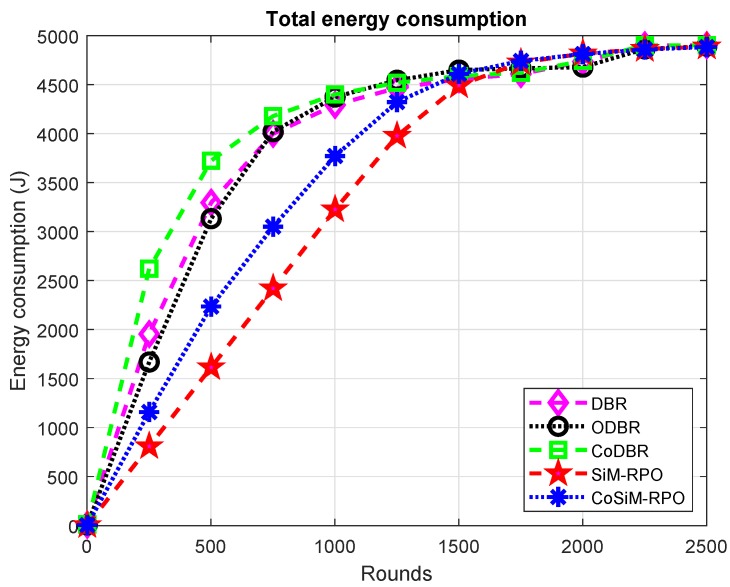
Consumption of energy.

Figure 6 indicates the cost paid as regards consumed energy. The energy consumed in the proposed schemes is the lowest than existing schemes. In the proposed SiM-RPO algorithm, the MSs moves in the network for the collection of the data, and direct transmission is done between the source and MS. Due to the direct transmission of the data with MSs, it reduces the energy cost of the network, and almost only one node involves in data exchanging, which leads to less energy consumption, while in the CoSiM-RPO, two nodes involve in data forwarding due to which more energy is consumed than the SiM-RPO algorithm, while in DBR, ODBR, and CoDBR, data delivery through multiple hops for a single packet to the sink is used. Multiple nodes involve in data transmission, which leads to extra energy being consumed, and as a result, the network has a greater energy consumption than the proposed SiM-RPO algorithm.

The consumed energy of CoSiM-RPO is greater than SiM-RPO due to the cooperative technique. The relay node transmits data to the MS when it receives a request from the MS, which leads to excessive energy consumption, while the energy expenditure of the CoSiM-RPO is the lowest among the existing algorithms. It is due to the involvement of many nodes for single-packet delivery in the existing schemes, while in the CoSiM-RPO, at most two nodes are involved in data transmission, leading to less consumption of energy comparatively.

ODBR has the lowest energy consumption with respect to DBR and CoDBR. ODBR balances the cost of energy by assigning energy in an organized manner based on data load on the nodes, while in DBR, all nodes have the same energy level. Therefore, ODBR’s energy cost is lower than DBR and CoDBR, because CoDBR consumes a high amount of energy due to the greater number of nodes involve in data forwarding.

CoDBR has the greatest energy consumption among the other algorithms. In CoDBR, the data are cooperatively forwarded to the destination. Almost one packet is delivered to the destination along three paths, and three nodes are involved in a single delivery. Due to the cooperation of the nodes, they consume the greatest energy, while in DBR and ODBR, the data are delivered over a single path through multiple hops to the sink and energy is consumed only by one node for a single-packet transmission to the next forwarder. Therefore, the energy consumed by DBR and ODBR is lower than CoDBR. The cost paid as consumed energy is summarized as SiM-RPO energy consumption < CoSiM-RPO < ODBR < DBR < CoDBR.

**Figure 7:**
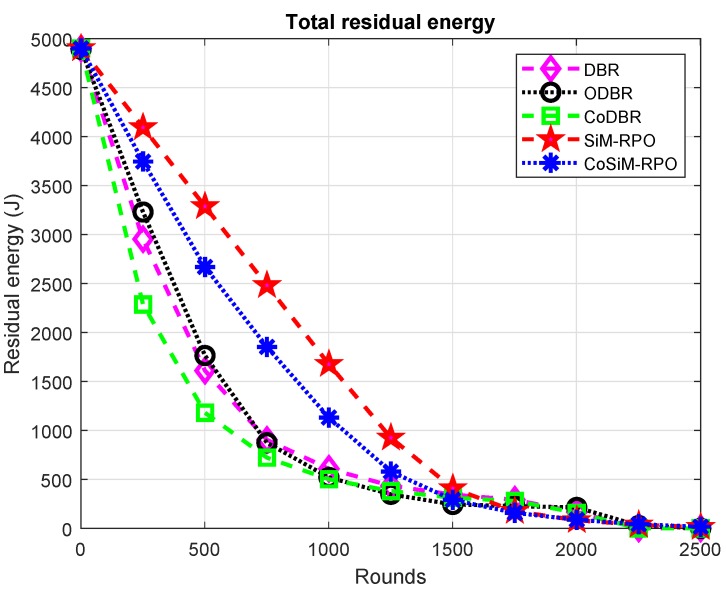
Remaining energy.

The remaining energy is reciprocally related to the energy cost. Therefore, all the above discussion for the consumption of energy is conversely true for the residual energy and is shown in Figure 7.

**Figure 8:**
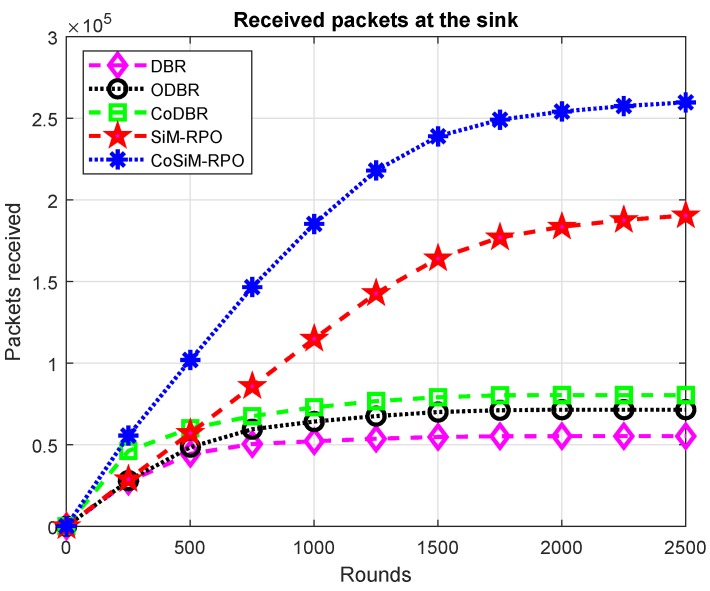
Received packets.

The CoSiM-RPO algorithm outperformed all the schemes in terms of packets reception, as shown in Figure 8. The CoSiM-RPO algorithm has the greatest packet reception. In the proposed algorithm, MSs move to establish a direct link between the node and themselves for data collection directly from the nodes without multiple hops, which enhances the chance of the packets’ reception. Furthermore, the MS checks the data quality to receive accurate data. If the received data do not meet the specified criteria of correct reception, then a request for retransmission is sent, and the relay forwards the data again toward the MS. Thus, this results in more packets being received at the MSs. While in the SiM-RPO scheme, the data are delivered only in a single path to the MS having a greater chance of packets being dropped compared to the CoSiM-RPO, the packets’ reception is the highest in CoSiM-RPO. In the other counterpart schemes, the data are delivered through multiple relays to the sinks. Therefore, there is a high probability of data destruction due to the channel effects, and the chance of the packets’ reception is always lower than the proposed scheme. Furthermore, the network density is directly related to the packet reception. This is because, if the nodes died, then the path to the sink is broken, and all the packets is dropped.

The SiM-RPO algorithm has the greatest packet reception compared to the counterpart schemes. This is because the packet is delivered directly to the MS without multiple hops. The direct transmission has less chances of data corruption instead of multiple hops due to the channel effects. While in the counterpart schemes, the data are delivered in a multi-hop fashion, the packets reception in the SiM-RPO algorithm is the highest among the counterpart schemes. While before 500 rounds, the SiM-RPO has lower packets reception than CoDBR, this is because the data reception probability is high due to cooperation in CoDBR.

The packet reception of CoDBR is the greatest compared to DBR and ODBR. The CoDBR algorithm forwards data to the destination along three different paths to reduce the channel effects. Therefore, the chance of the packets’ reception is higher in CoDBR than in DBR and ODBR, while in DBR and ODBR, a single path that may be noisy and have greater channel effects is followed toward the sink.

More packets are received in ODBR than DBR. This is due to data load balancing by assigning different energy, which leads to greater packets reception in ODBR. Thus, reception of the data in DBR is the lowest compared to the other schemes. The packets’ reception in all of the schemes is in the order: CoSiM-RPO packets’ reception > SiM-RPO > CoDBR > ODBR > DBR.

**Figure 9:**
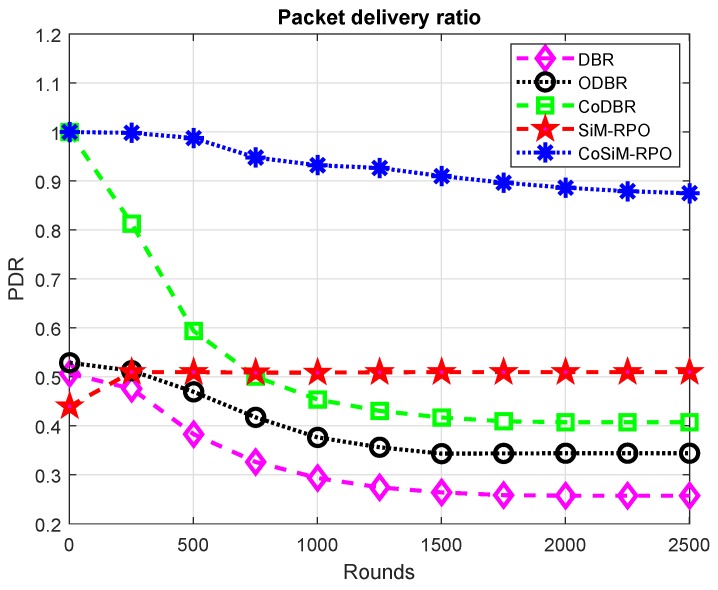
PDR.

PDR is highlighted in Figure 9. The PDR of the CoSiM-RPO algorithm is the best compared to the other schemes. This is due to forwarding of the data in a cooperative manner to the MSs directly. The direct transmission of the data in a cooperative manner enhanced the PDR due to high packet reception in the CoSiM-RPO. The CoDBR also forwards data cooperatively to the sink, but the delivery of the data to the sink is done through other nodes, which reduces the packets’ reception at the sink and tends toward a lesser PDR. The other schemes DBR, ODBR, and SiM-RPO are non-cooperative algorithms, which is the reason that the PDR is the lowest.

The PDR of the proposed scheme is the best compared to the other existing algorithms, except CoDBR, up to 750 rounds. This is because, at the start, more nodes are available and have a greater chance of cooperation among them in CoDBR, which tends to higher PDR. After 750 rounds, due to the death of the nodes chances of cooperative data forwarding reduce, which makes the PDR go below the PDR of SiM-RPO. In the SiM-RPO algorithm, reception of the data at the MSs is the highest due to the direct exchanging of the data between the node and sink tending to get the highest PDR among the other counterpart schemes. The behavior of the PDR of the SiM-RPO scheme is almost constant in every round. This is because it has no dependency on the other nodes in the network during transmission of the data between the sender and sink. The MSs try to reach every node to collect data directly.

The PDR of the CoDBR algorithm has the greatest compared to the DBR and ODBR schemes. CoDBR has greater packet reception and less probability of packets being dropped due to the cooperation of the nodes. Therefore, CoDBR achieves higher PDR, while in DBR and ODBR, only a single path is followed toward the sink and has a greater probability of packets being dropped and less reception of data, which tends to achieve the lowest PDR. The PDR of ODBR is better than DBR due to more packets being received, while the DBR has the lowest PDR among the other schemes. The PDR is in the order: CoSiM-RPO > CoDBR > SiM-RPO > ODBR > DBR.

**Figure 10:**
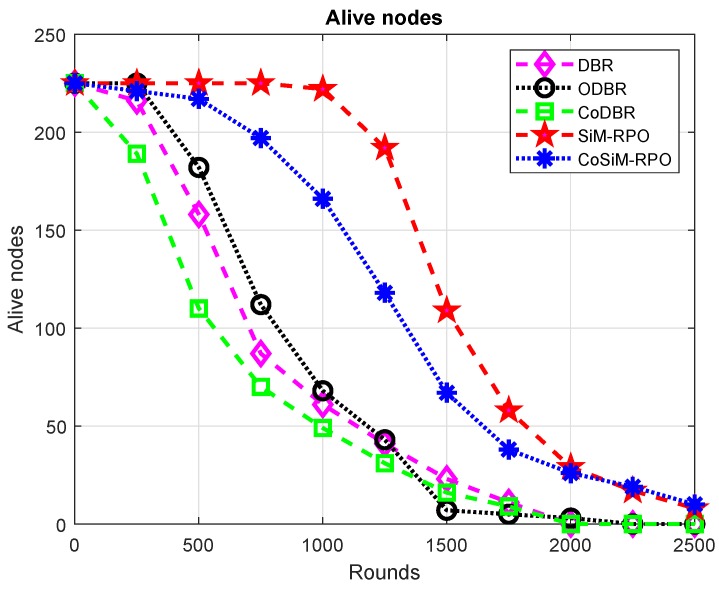
Number of alive nodes.

As highlighted in Figure 10, the number of alive nodes in the SiM-RPO algorithm is maximal. In the counterpart schemes, the data delivery through the multi-hop fashion leads to draining of the energy of a node at a high rate, which tends to die soon, while in the proposed SiM-RPO algorithm, the transmission between the source and sink is held through a direct path without any other relays, due to which, less energy consumes, and nodes are alive for the longest period of time. Hence, the proposed SiM-RPO has the maximum alive nodes with respect to the other algorithms, while in CoSiM-RPO, the data is delivered also by the relay to the MSs in the case of failure. Therefore, the number of alive nodes become greater than SiM-RPO.

The number of alive nodes in the CoSiM-RPO algorithms is the highest compared to the other existing schemes, because in the proposed cooperative scheme, the MSs tries to reach every node for the collection of the data directly, which reduce the energy expenditure and enhanced the lifetime of the network, resulting the nodes to be alive for more time, while in the existing schemes, the packet delivery is done through many other nodes to the sink, which leads to extra energy being consumed and having a lesser number of alive nodes.

ODBR has more alive nodes than DBR and CoDBR, because the data is forwarded in a single path to the sink and it manages the energy distribution between the nodes. The energy distribution leads to greater network lifetime; hence, the nodes are alive for a long time as compared to the DBR and CoDBR schemes.

**Figure 11:**
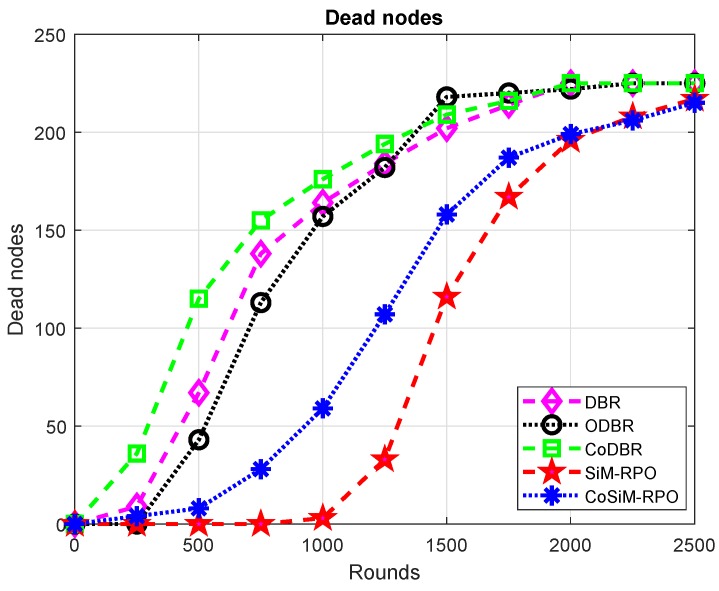
Number of dead nodes.

DBR has more alive nodes than CoDBR. In DBR, only a single node is used for data forwarding, which consumes less energy and tends to be alive for more time, while in CoDBR, due to cooperation for a single packet transmission, three nodes are required, which drained the energy at a high rate, and they tend to die earlier than DBR. The aforesaid discussion for the alive nodes is conversely true for the number of dead nodes, as shown in Figure 11. The number of alive nodes is in the order: SiM-RPO > CoSiM-RPO > ODBR > DBR > CoDBR.

**Figure 12:**
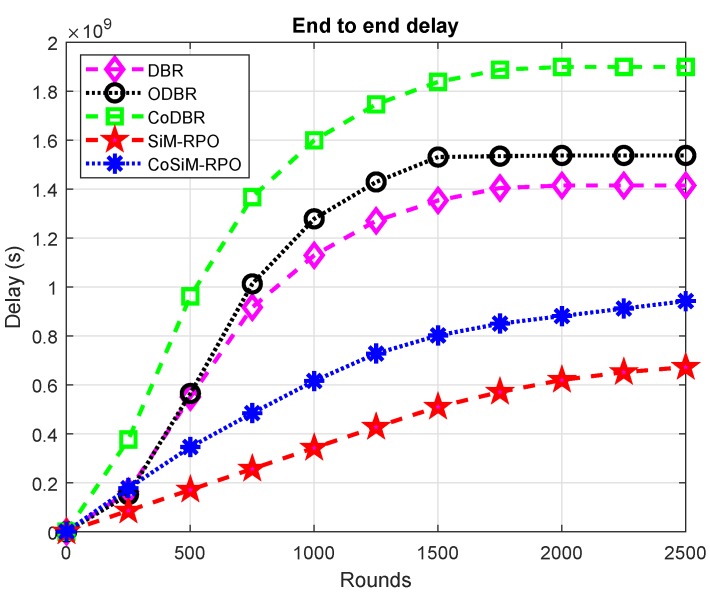
Latency of the network.

The latency of all the algorithms is demonstrated in Figure 12. CoDBR has the greatest latency compared to all the other schemes. This is due to the data reception at the final destination through multiple paths using cooperation, which takes time to combine the data received from different forwarders, while in CoSiM-RPO, the data is forwarded in a cooperative manner directly to the MS, which takes less time compared to CoDBR. The other schemes are non-cooperative, transmitting data through a single path, leading to less time consumption.

The latency of ODBR is greater than DBR, SiM-RPO, and CoSiM-RPO. This is due to the many eligible forwarders in ODBR, which consumes time to deliver data to the final destination, while in DBR, the nodes at the surface die soon and has a fewer number of eligible forwarders, tending to consume less time. In the proposed schemes, the delivery is done directly without any relaying data, which reduces the latency.

DBR has the greatest delay compared to the proposed schemes due to the multiple paths routing, while in the proposed scheme, information is exchanged directly, which is delivered in less time. Time comparison between the proposed schemes shows that CoSiM-RPO transfers data with a greater amount of time compared to SiM-RPO. This is due to the retransmission of the data in CoSiM-RPO, which takes time, while there is no retransmission in the SiM-RPO algorithm. The latency is in the order: CoDBR > ODBR > DBR > CoSiM-RPO > SiM-RPO.

## 7. Conclusions and Future Insights

The proposed SiM-RPO and CoSiM-RPO algorithms are presented for the reliable and persistence operation of the network. For the MSs’ path determination, the network is split into four equal squares. To cover all the network and collect more information, the triangular paths are defined for the MSs. The MSs move in the network to pick information and data directly from the nodes. In CoSiM-RPO, for correct data delivery, the data are determined at the MSs. If the data satisfy the criteria of the correct data reception, then this is considered successful delivery of the data. Otherwise, the MSs demands retransmission of the data from the relay, which enhances the probability of correct data reception. The proposed algorithm enhances the performance of the UA-WSNs in terms of successful data reception with minimum energy cost and delay and has more stability than the existing counterpart algorithms. In the future, energy harvesting techniques will be utilized for further improvement of the network stability.

## Figures and Tables

**Table 1:** Parameters.

S.No	Parameter	Value
1	Network size	500 m × 500 m
2	Number of nodes	225
3	Sink nodes	4
4	Initial energy	20 joules
5	Transmission range	130 m
6	Frequency	30 kHz
7	Data packet size	1600 bits
